# Supplementary material for: Structure and function of the ROR2 cysteine-rich domain in vertebrate noncanonical WNT5A signaling
Source: eLife. 2024 May 23;13:e71980. doi: 10.7554/eLife.71980 (PMC11219042; doi:10.7554/eLife.71980)
Supplement: Supplementary file 2. — APDB accession codes displayed in parenthesis; BRMSD (root mean square deviation) values were calculated for equivalent Cα atom positions using the program SHP (Stuart et al., 1979; Riffel et al., 2002); CNumber of equivalent Cα positions used in calculation of RMSD values with SHP; DSummed structural correlation (total probability) values calculated via SHP. The phylogenetic tree for CRDs analyzed (Figure 1C) was assembled using PHYLIP (Felsenstein, 1989). These summed structural correlation values were used to construct a distance matrix. FZ8-PAM – Frizzled 8-palmitoleate complex (Janda et al., 2012), Smo – Smoothened (Byrne et al., 2016), FZ8 – Frizzled 8 (Dann et al., 2001), sFRP3 – secreted Frizzled-related protein 3 (Dann et al., 2001), MuSK – muscle-specific kinase (Stiegler et al., 2009), NPC1 – Niemann-Pick C1 protein (Kwon et al., 2009), RFBP – riboflavin-binding protein (Monaco, 1997), FRα – folate receptor α (Chen et al., 2013), FRβ – folate receptor β (Wibowo et al., 2013), JUNO – folate receptor δ (White et al., 2016). FZ7 – Frizzled 7 (Nile et al., 2017). [file elife-71980-supp2.docx]

**Supplementary Table 2**

| **Protein** | **Fz8-PAM**  **(4F0A)**^A^ | **Smo (5L7D)** | **Fz8**  **(1IJY)** | **sFRP3 (1IJX)** | **MuSK (3HKL)** | **NPC1**  **(3GKI)** | **RFBP (not in PDB)** | **FRα (4LRH)** | **JUNO (5EJN)** | **FRβ (4KMZ)** |
| --- | --- | --- | --- | --- | --- | --- | --- | --- | --- | --- |
| **ROR2** | 1.19^B^  81^C^  39.62^D^ | 1.30  78  35.87 | 1.30  83  37.19 | 1.28  83  36.53 | 0.88  110  62.08 | 1.94  74  27.99 | 2.40  58  17.20 | 2.50  54  15.39 | 2.43  50  15.10 | 2.38  51  15.57 |
| **Fz8-PAM (4F0A)** |  | 1.00  83  43.64 | 0.16  117  102.54 | 0.42  106  79.01 | 1.30  78  38.33 | 2.17  61  21.36 | 2.54  40  13.35 | 2.76  44  12.16 | 2.51  44  13.31 | 2.44  43  14.14 |
| **Smo (5L7D)** |  |  | 1.09  84  43.18 | 1.04  80  44.60 | 1.26  78  40.84 | 2.32  54  19.01 | 2.68  46  11.24 | 2.62  46  13.73 | 2.64  44  12.47 | 2.49  48  15.65 |
| **Fz8 (1IJY)** |  |  |  | 0.45  108  78.07 | 1.35  76  36.13 | 2.14  62  22.27 | 2.50  45  15.00 | 2.66  43  13.41 | 2.38  45  15.40 | 2.53  43  14.34 |
| **sFRP3 (1IJX)** |  |  |  |  | 1.36  79  36.65 | 2.41  60  17.28 | 2.31  45  17.60 | 2.59  47  14.13 | 2.49  39  13.87 | 2.47  46  15.62 |
| **MuSK (3HKL)** |  |  |  |  |  | 2.14  67  22.48 | 2.25  55  20.03 | 2.30  55  15.14 | 2.16  53  19.21 | 2.13  53  23.24 |
| **NPC1 (3GKI)** |  |  |  |  |  |  | 1.73  82  35.46 | 1.95  89  31.57 | 1.74  81  36.37 | 1.74  90  30.58 |
| **RFBP (not in PDB)** |  |  |  |  |  |  |  | 0.68  160  104.06 | 0.85  138  80.30 | 0.66  157  105.39 |
| **FRα (4LRH)** |  |  |  |  |  |  |  |  | 0.42  161  125.21 | 0.16  194  176.05 |
| **JUNO (5EJN)** |  |  |  |  |  |  |  |  |  | 0.46  157  120.83 |
