# Supplementary material for: Structure and function of the ROR2 cysteine-rich domain in vertebrate noncanonical WNT5A signaling
Source: eLife. 2024 May 23;13:e71980. doi: 10.7554/eLife.71980 (PMC11219042; doi:10.7554/eLife.71980)
Supplement: Source data 1. [file elife-71980-data1.zip › Original and uncropped gel and blot images/Figure 4-figure supplement-uncropped blots with labels.pdf]

Figure 4-figure supplement
Uncropped blots with lables

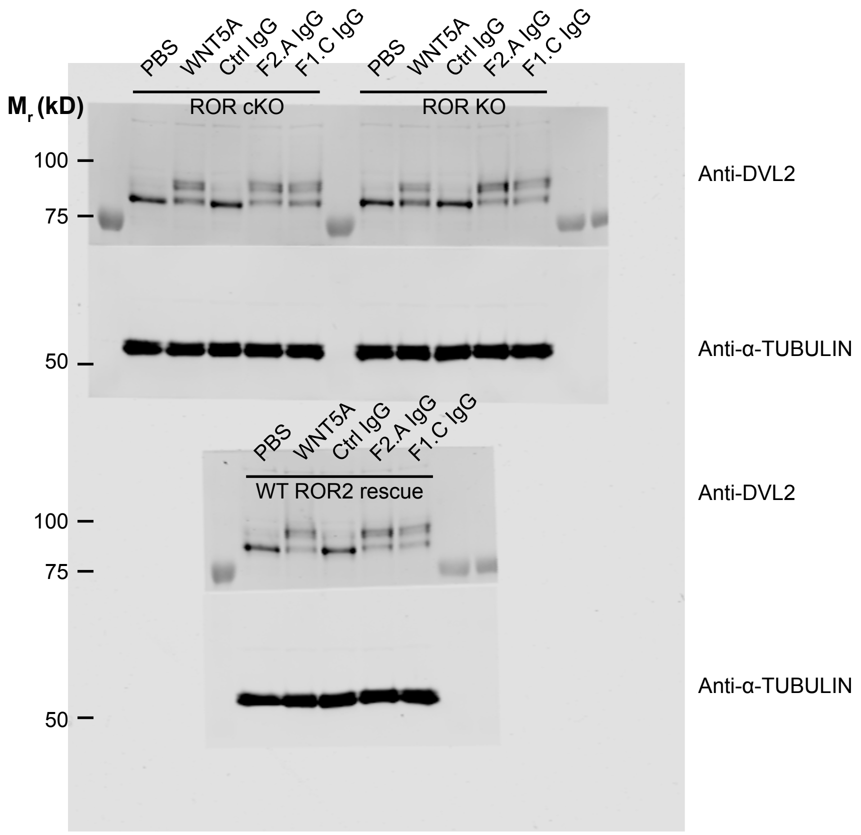

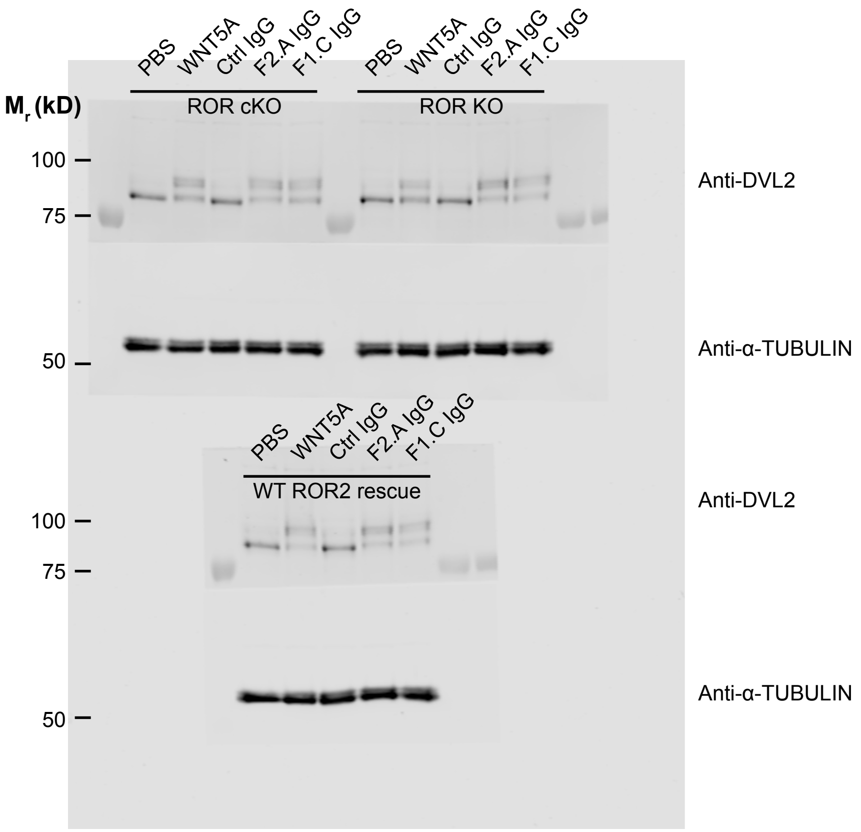

Top blots used for the DVL2 panels in the figure supplement
Bottom blots used for TUBULIN panels in the figure supplement
