# Supplementary material for: Structure and function of the ROR2 cysteine-rich domain in vertebrate noncanonical WNT5A signaling
Source: eLife. 2024 May 23;13:e71980. doi: 10.7554/eLife.71980 (PMC11219042; doi:10.7554/eLife.71980)
Supplement: Source data 1. [file elife-71980-data1.zip › Original and uncropped gel and blot images/Figure 4A-uncropped blot with labels.pdf]

Figure 4A
Uncropped blot with lables

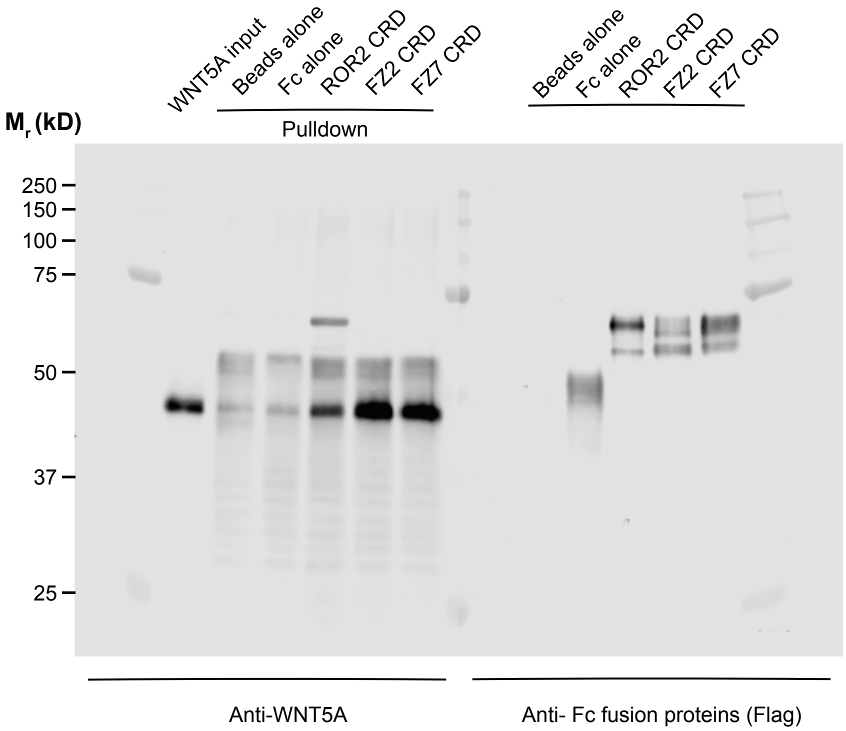

***The upper band in the ROR2 CRD lane of the anti-WNT5A panel is from a cross-reactivity of the anti-WNT5A antibody toward the ROR2 CRD. In an independent experiment in which we blotted ROR2 CRD-Kr with the same anti-WNT5A antibody, we observed the same antibody cross-reactivity at the exact molecular weight of the ROR2 CRD-Kr protein.
