# Supplementary figures and images for: Structure and function of the ROR2 cysteine-rich domain in vertebrate noncanonical WNT5A signaling
Source: eLife. 2024 May 23;13:e71980. doi: 10.7554/eLife.71980 (PMC11219042; doi:10.7554/eLife.71980)

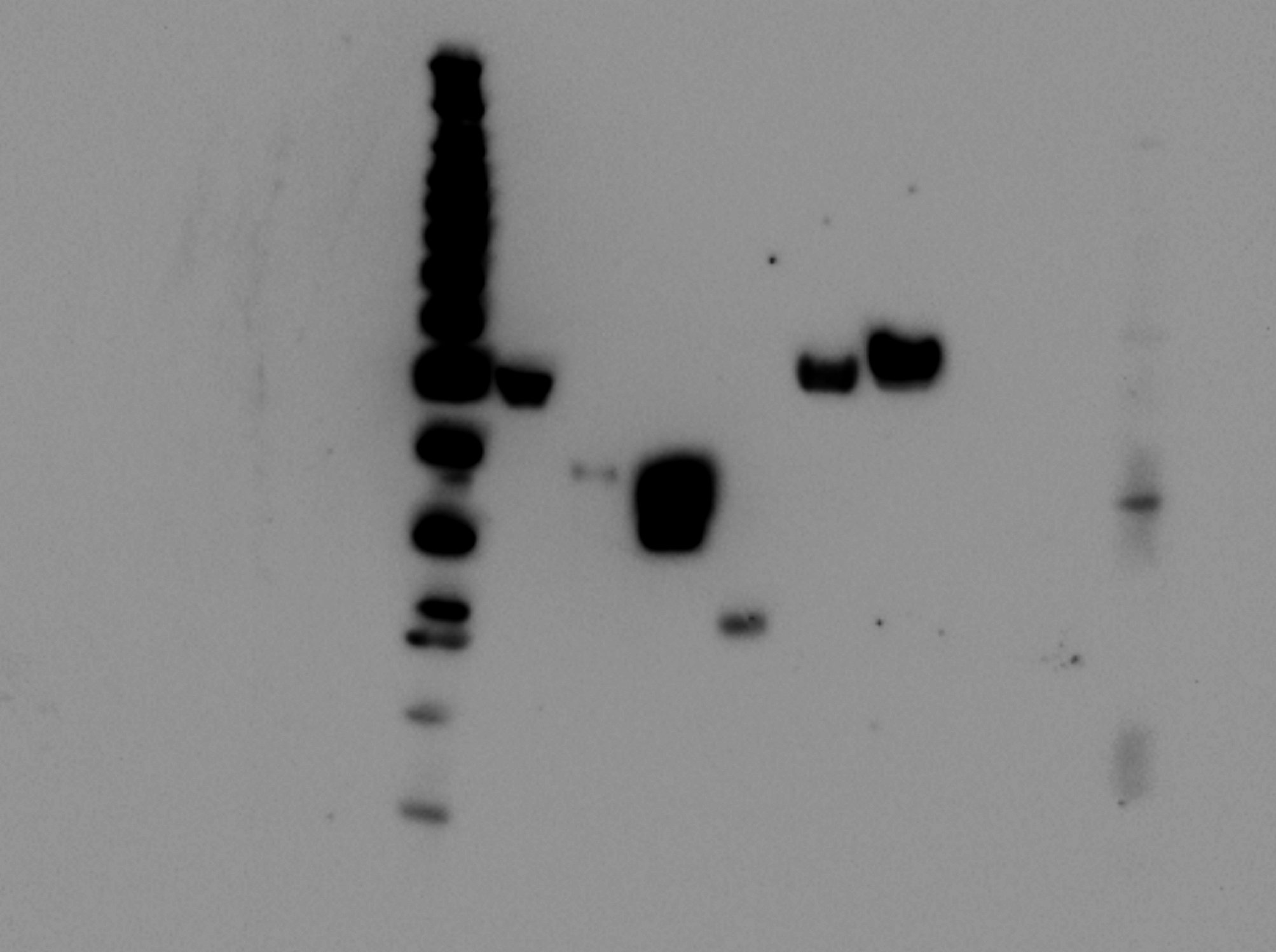

Supplement: Source data 1. [file elife-71980-data1.zip › Original and uncropped gel and blot images/Figure 1-figure supplement 1A-original blot.png]

Figure 1-figure supplement 1A  
Uncropped blot with labels

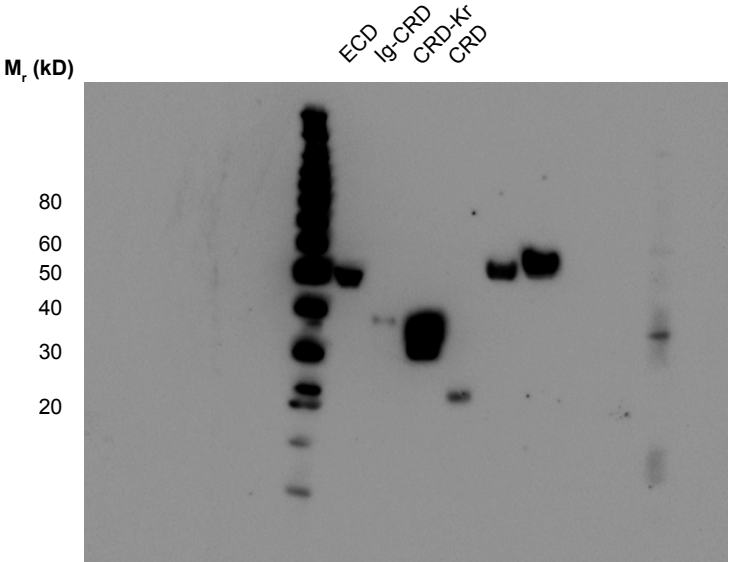

Supplement: Source data 1. [file elife-71980-data1.zip › Original and uncropped gel and blot images/Figure 1-figure supplement 1A-uncropped blot with labels.pdf]

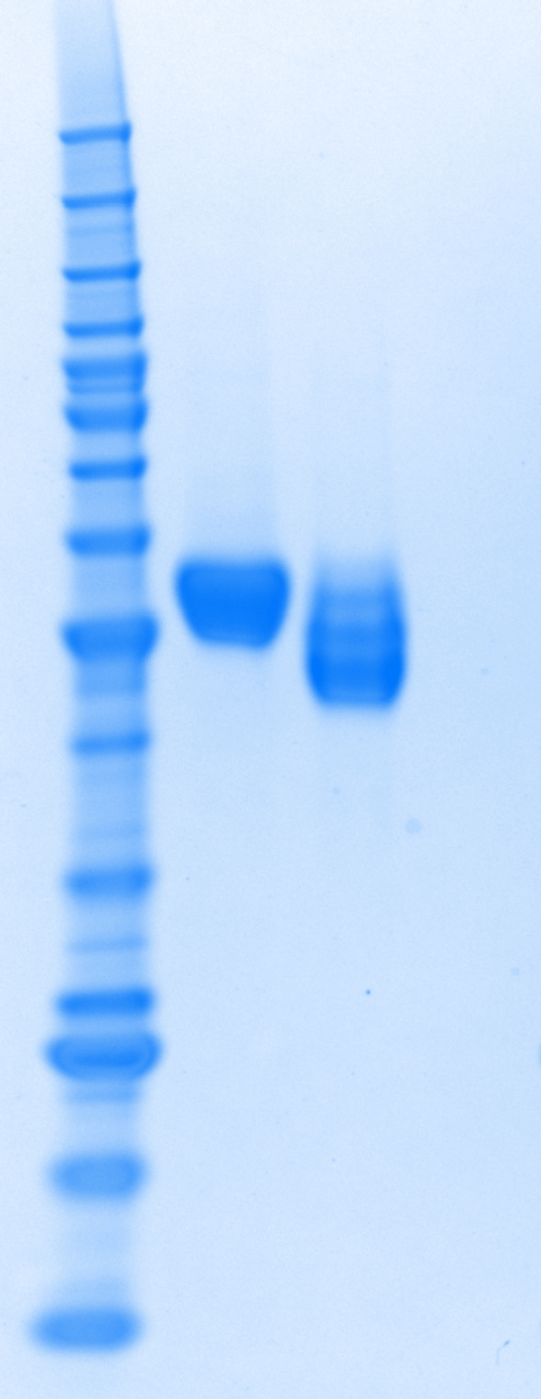

Supplement: Source data 1. [file elife-71980-data1.zip › Original and uncropped gel and blot images/Figure 1-figure supplement 1C-original gel.png]

Figure 1-figure supplement 1C  
Uncropped gel with labels

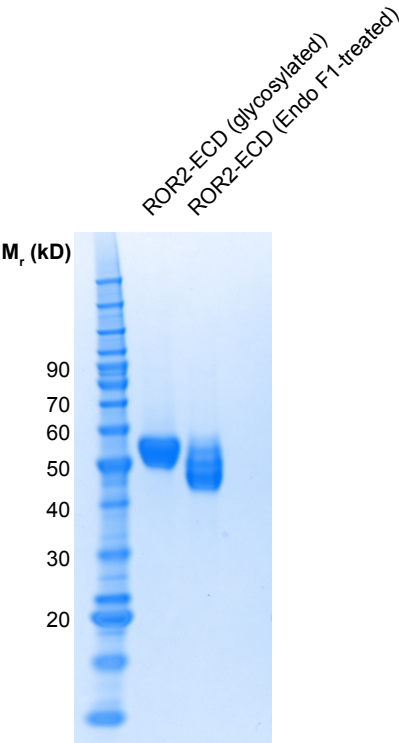

Supplement: Source data 1. [file elife-71980-data1.zip › Original and uncropped gel and blot images/Figure 1-figure supplement 1C-uncropped gel with labels.pdf]

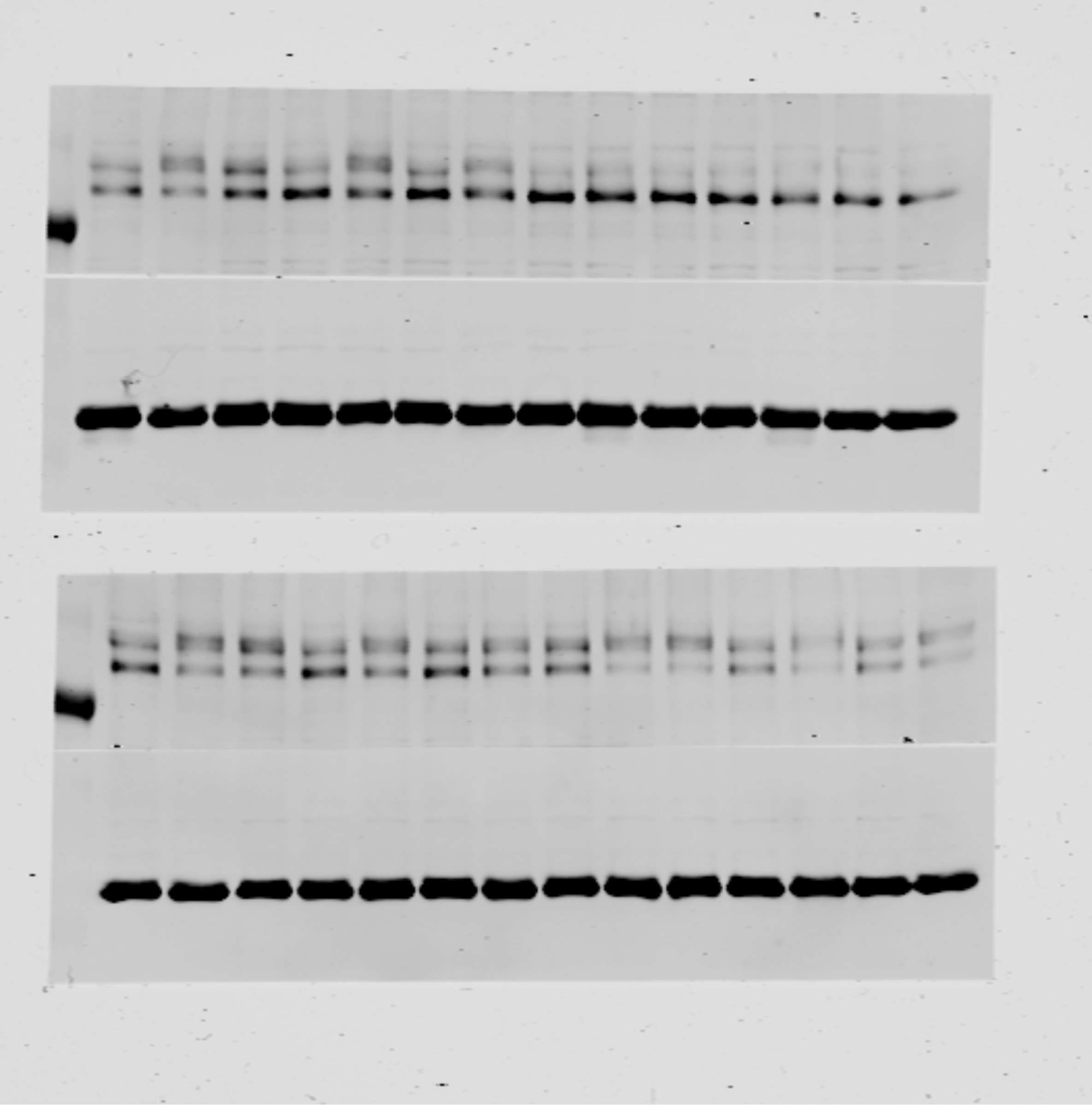

Supplement: Source data 1. [file elife-71980-data1.zip › Original and uncropped gel and blot images/Figure 3-figure supplement 2A-original blots used for DVL2 panels.png]

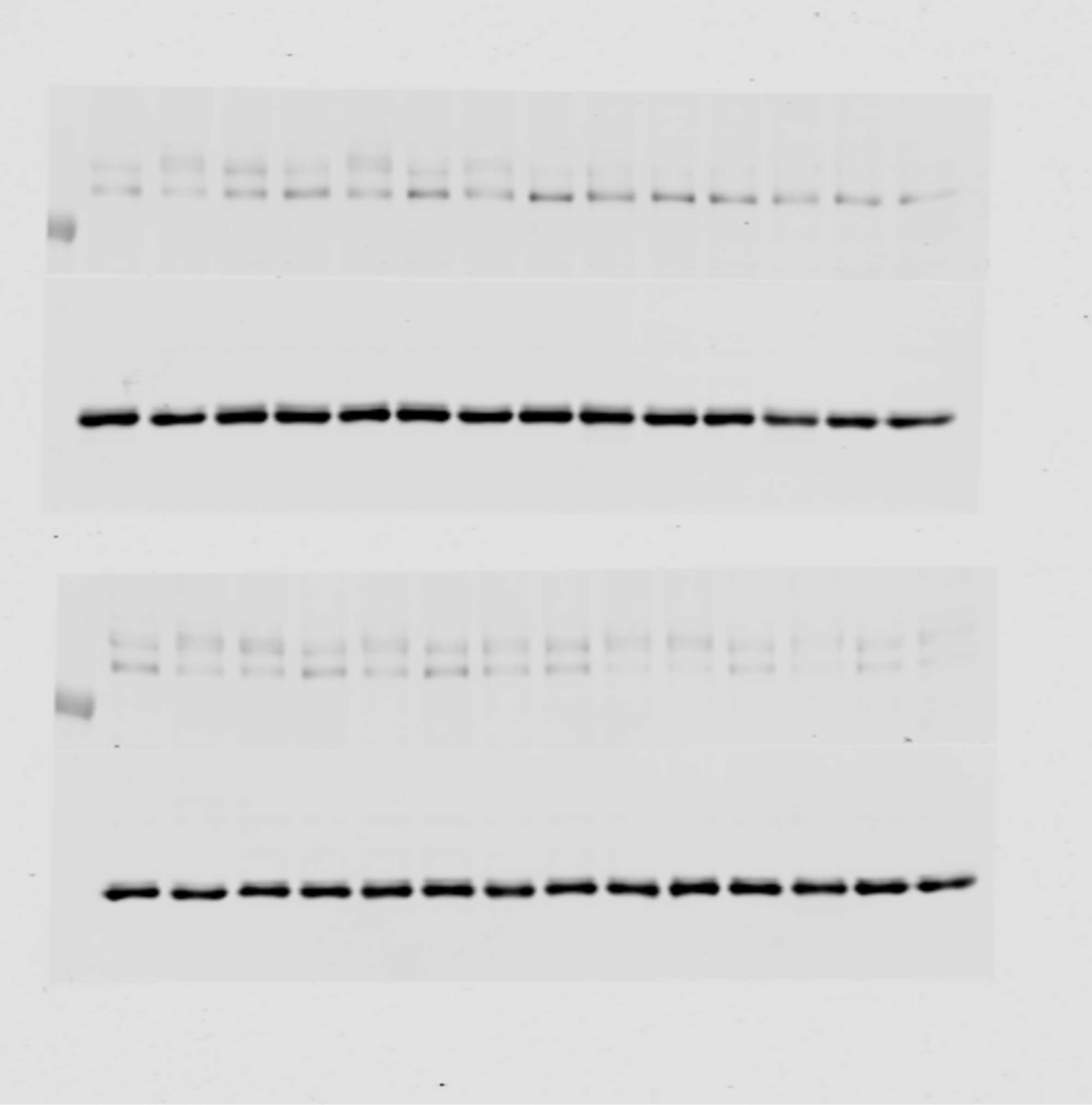

Supplement: Source data 1. [file elife-71980-data1.zip › Original and uncropped gel and blot images/Figure 3-figure supplement 2A-orignal blots used for tubulin panels.png]

Figure 3-figure supplement 2A  
Uncropped blots with labels

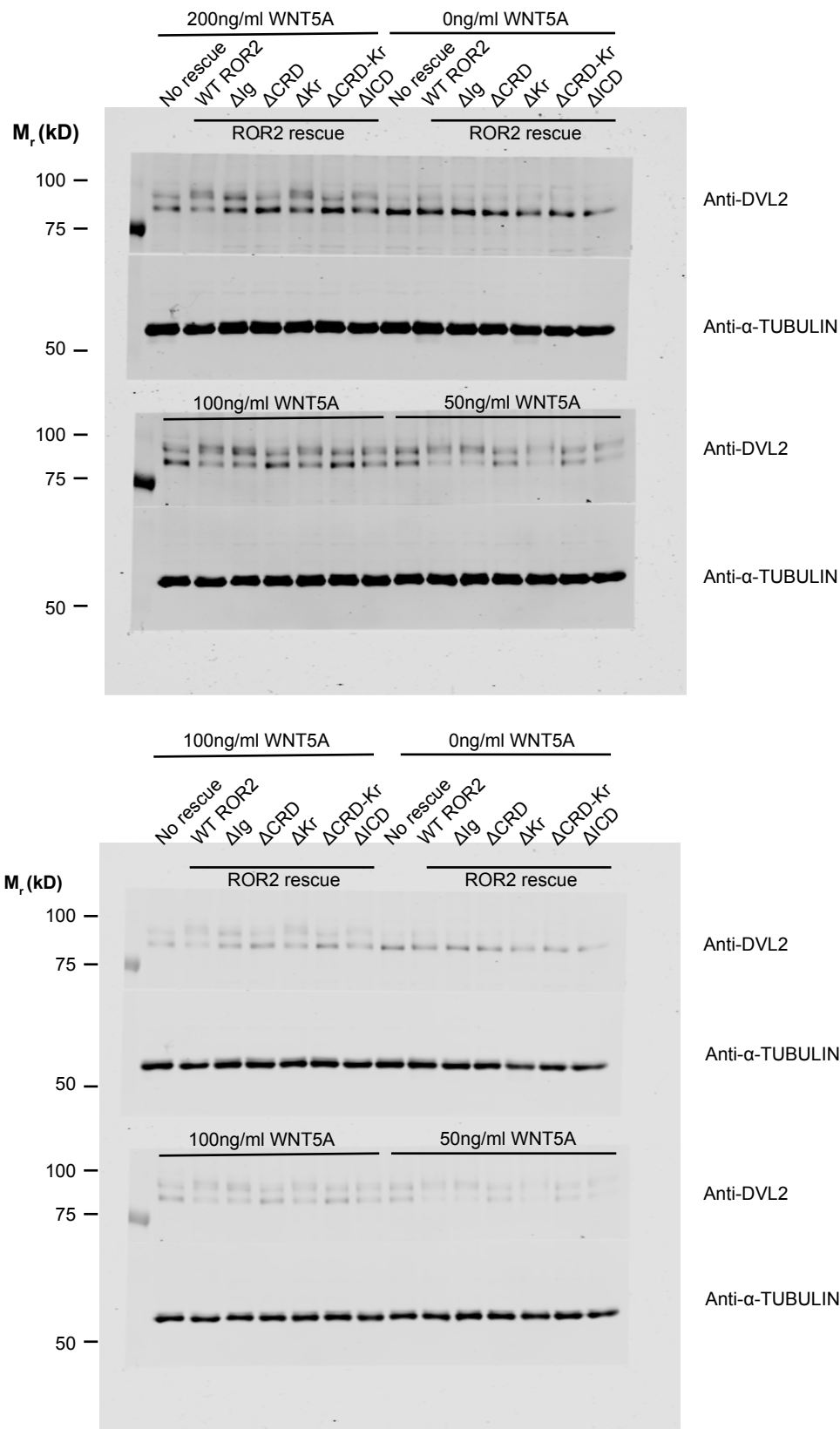

Supplement: Source data 1. [file elife-71980-data1.zip › Original and uncropped gel and blot images/Figure 3-figure supplement 2A-uncropped blots with labels.pdf]

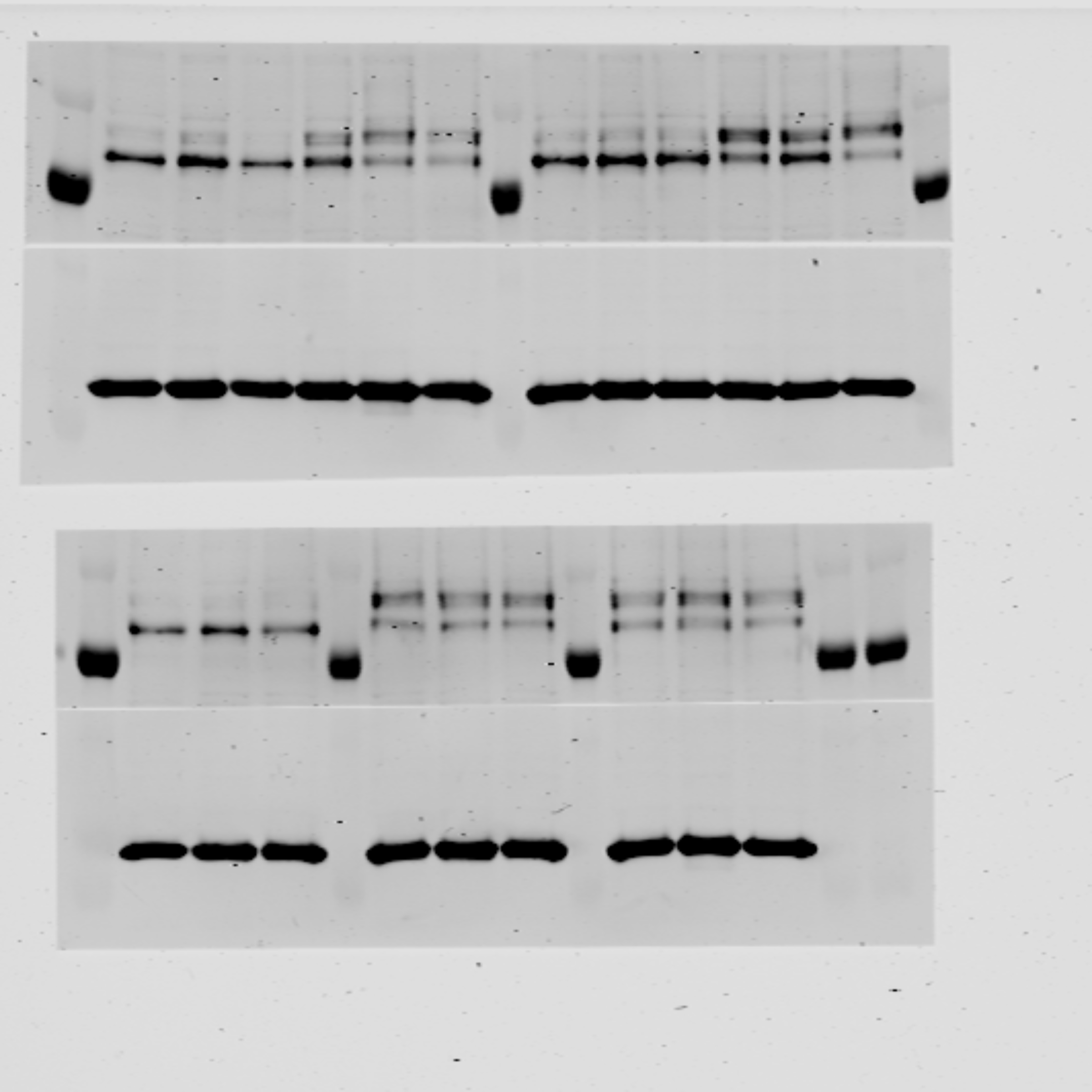

Supplement: Source data 1. [file elife-71980-data1.zip › Original and uncropped gel and blot images/Figure 3-figure supplement 2C-original blot used for DVL2 panel.png]

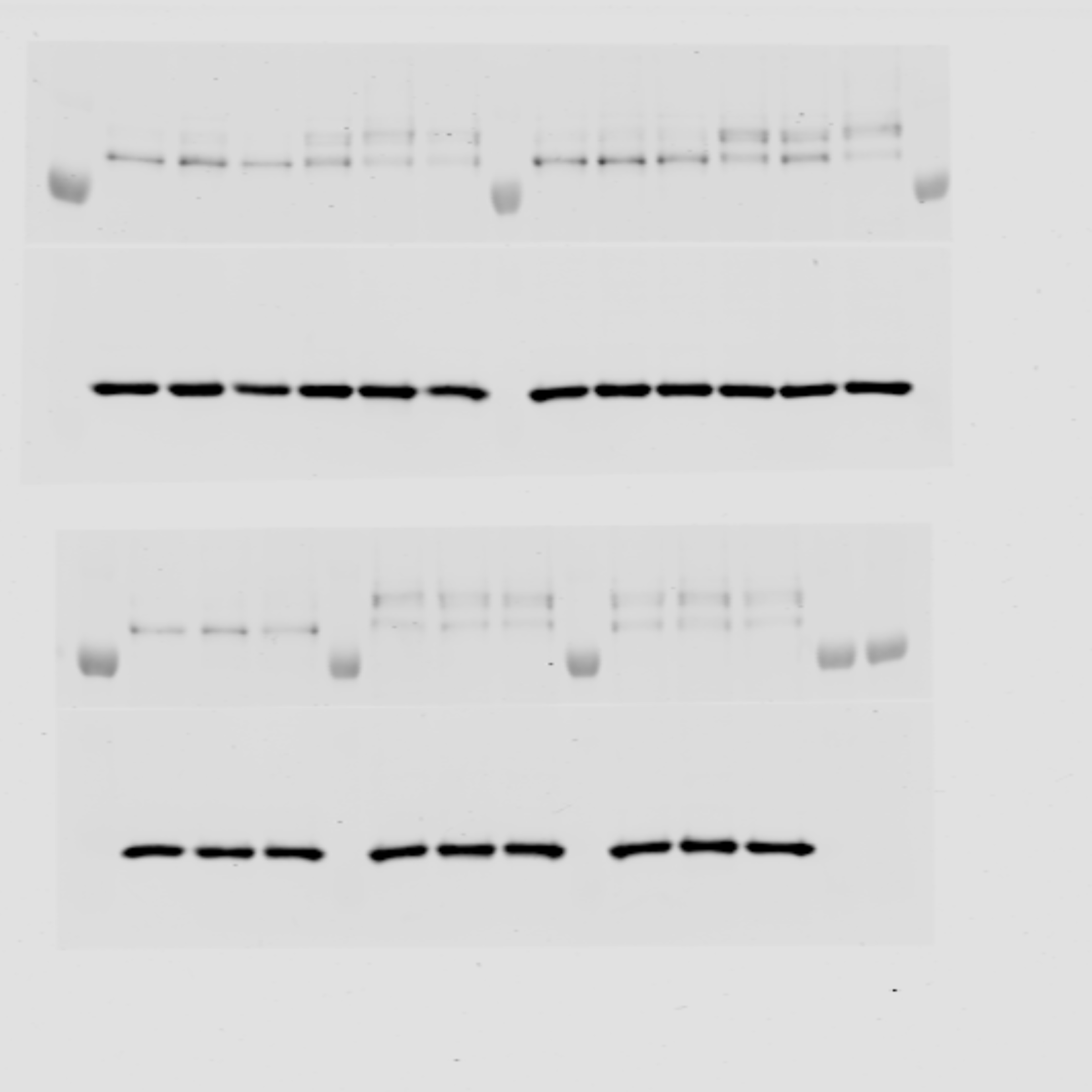

Supplement: Source data 1. [file elife-71980-data1.zip › Original and uncropped gel and blot images/Figure 3-figure supplement 2C-original blot used for tubulin panel.png]

Figure 3-figure supplement 2C  
Uncropped blots with labels

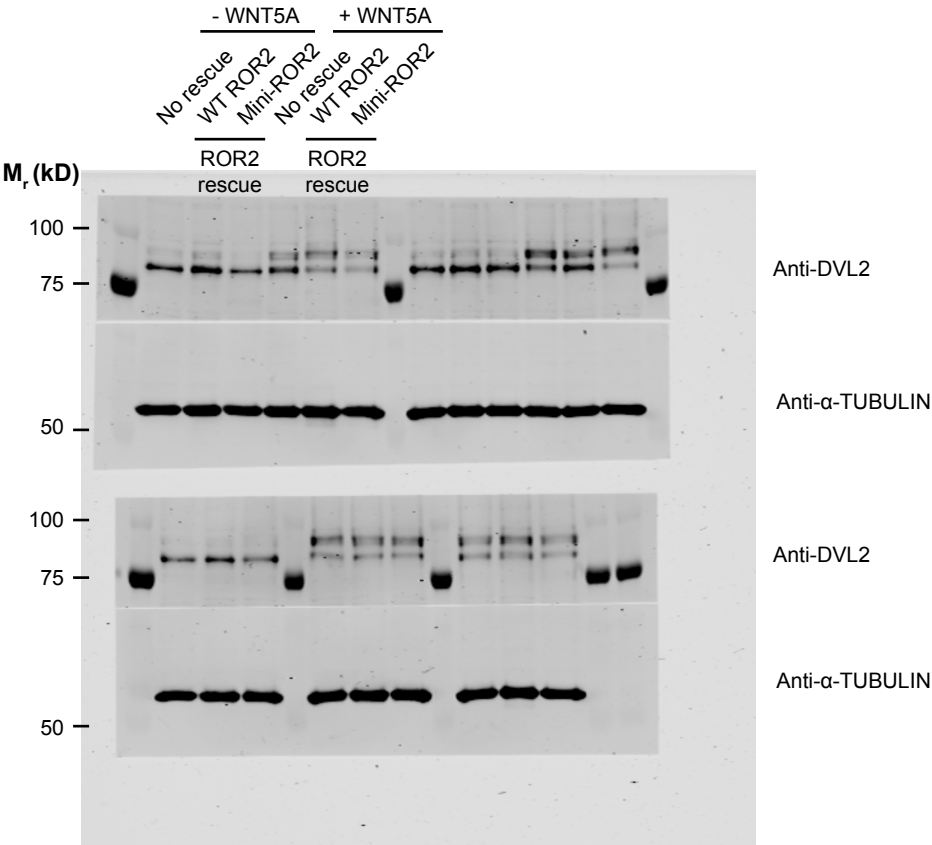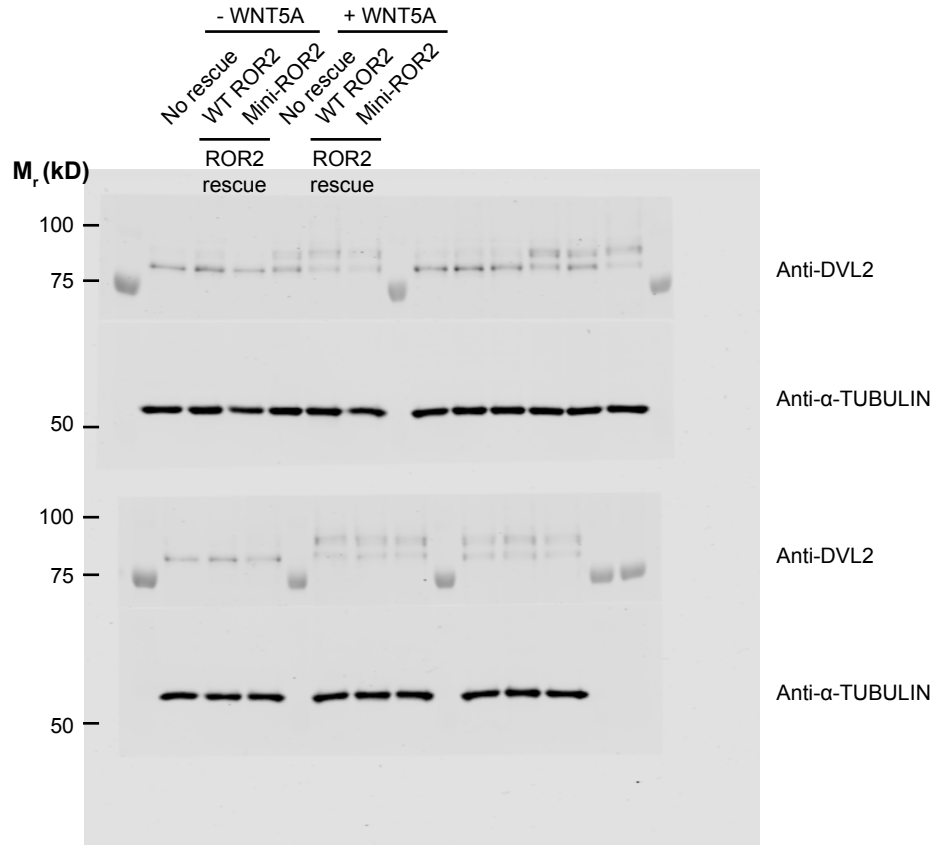

Supplement: Source data 1. [file elife-71980-data1.zip › Original and uncropped gel and blot images/Figure 3-figure supplement 2C-uncropped blots with labels.pdf]

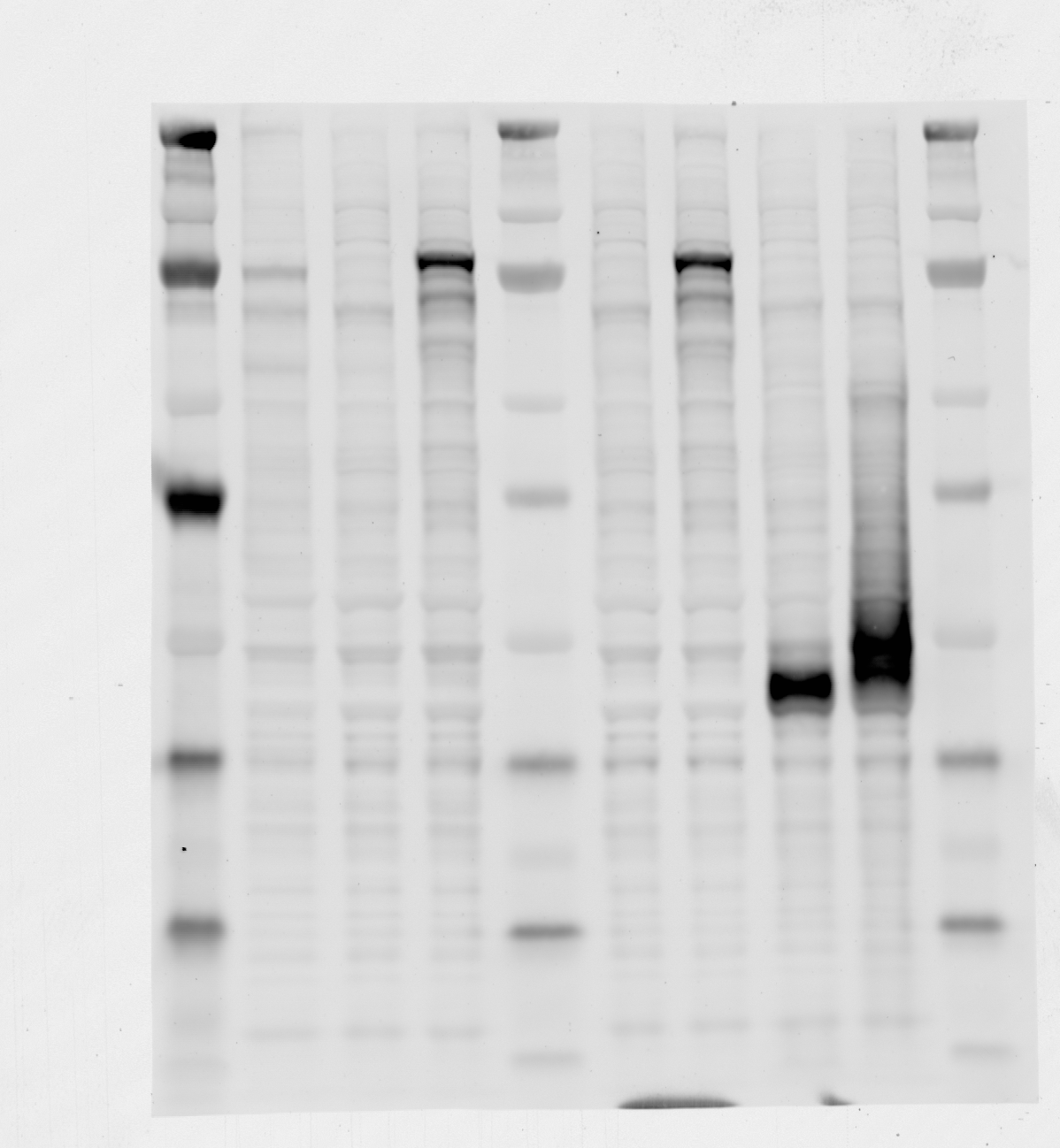

Supplement: Source data 1. [file elife-71980-data1.zip › Original and uncropped gel and blot images/Figure 3B-original Ror2 blot .png]

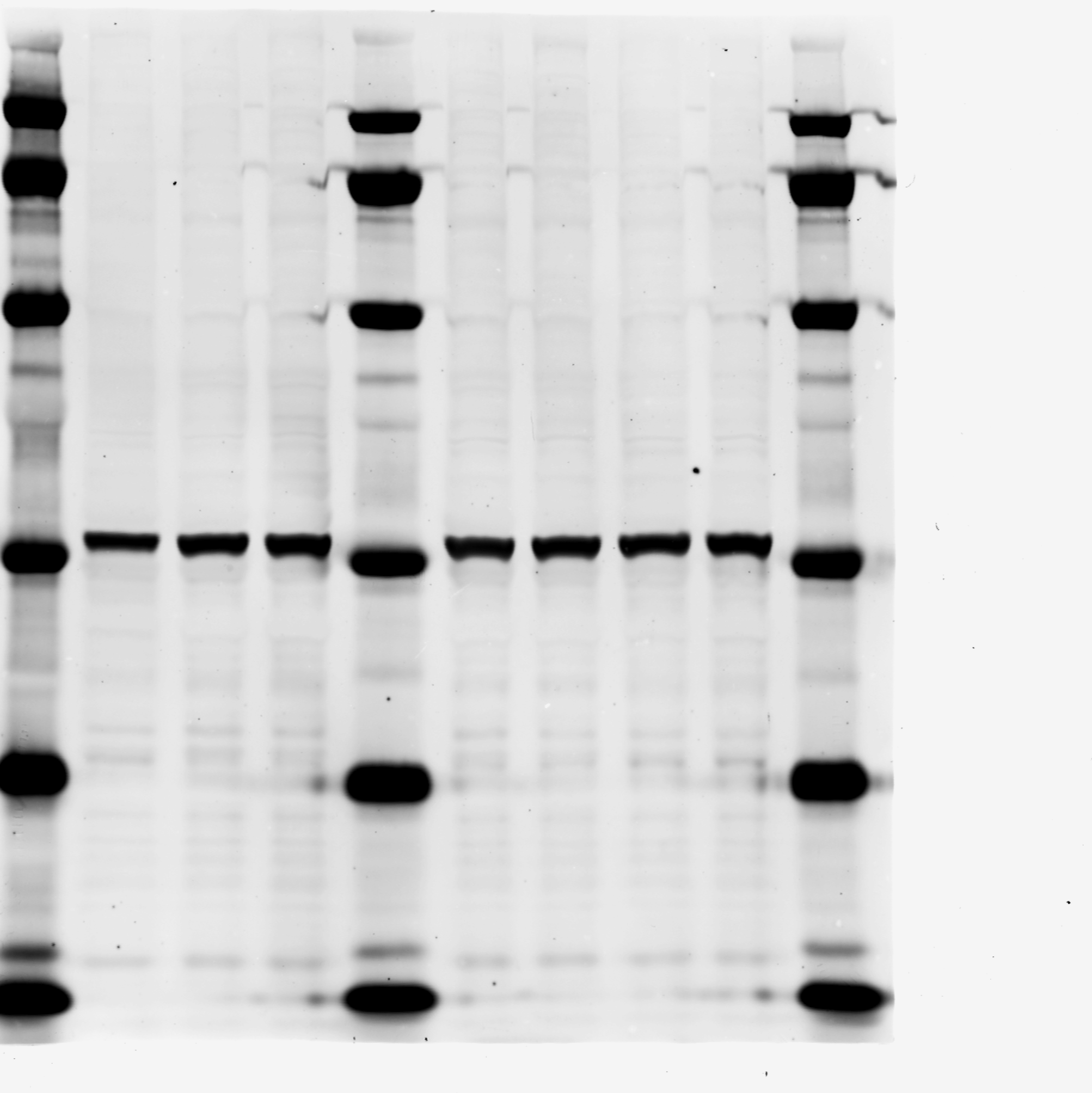

Supplement: Source data 1. [file elife-71980-data1.zip › Original and uncropped gel and blot images/Figure 3B-original tubulin blot.png]

**Figure 3B**  
**Uncropped blots with labels**

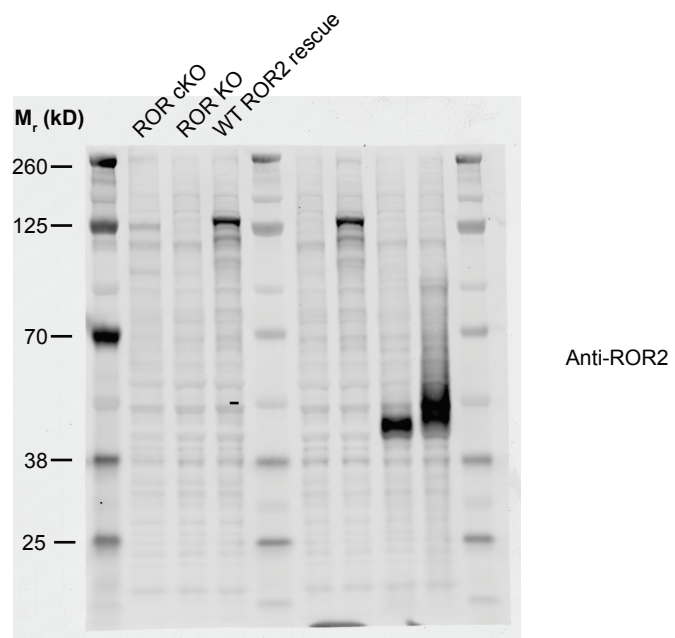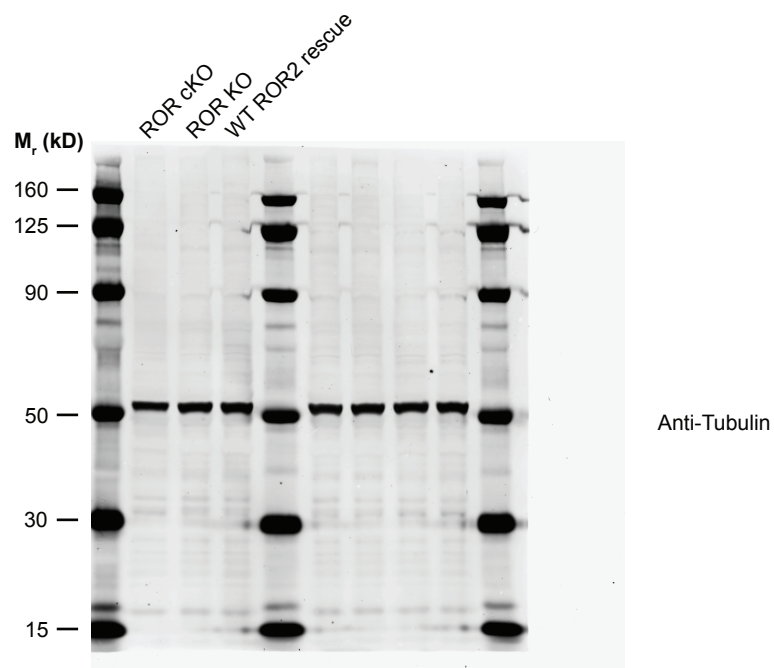

Supplement: Source data 1. [file elife-71980-data1.zip › Original and uncropped gel and blot images/Figure 3B-uncropped blots with labels.pdf]

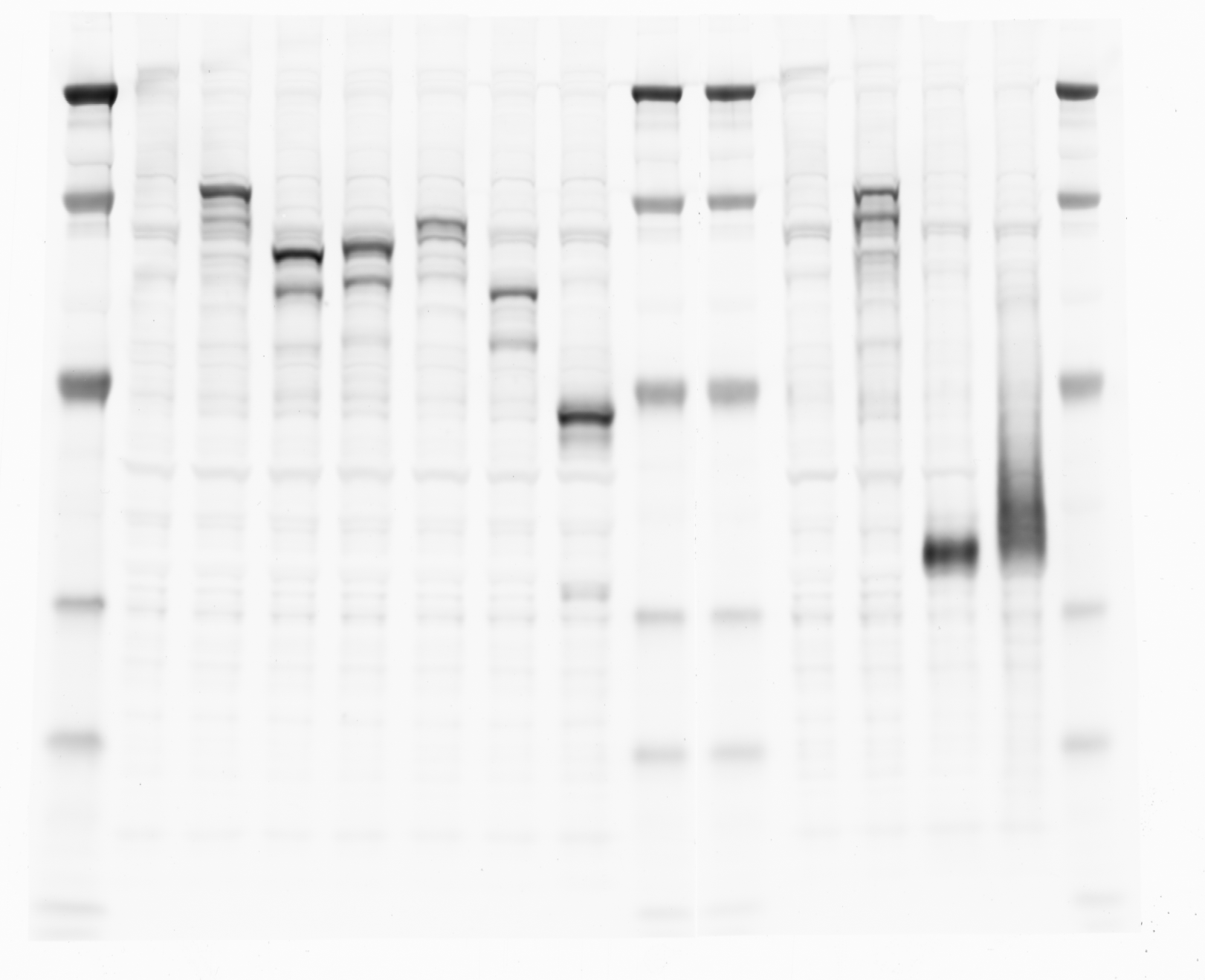

Supplement: Source data 1. [file elife-71980-data1.zip › Original and uncropped gel and blot images/Figure 3E-original Ror2 blot.png]

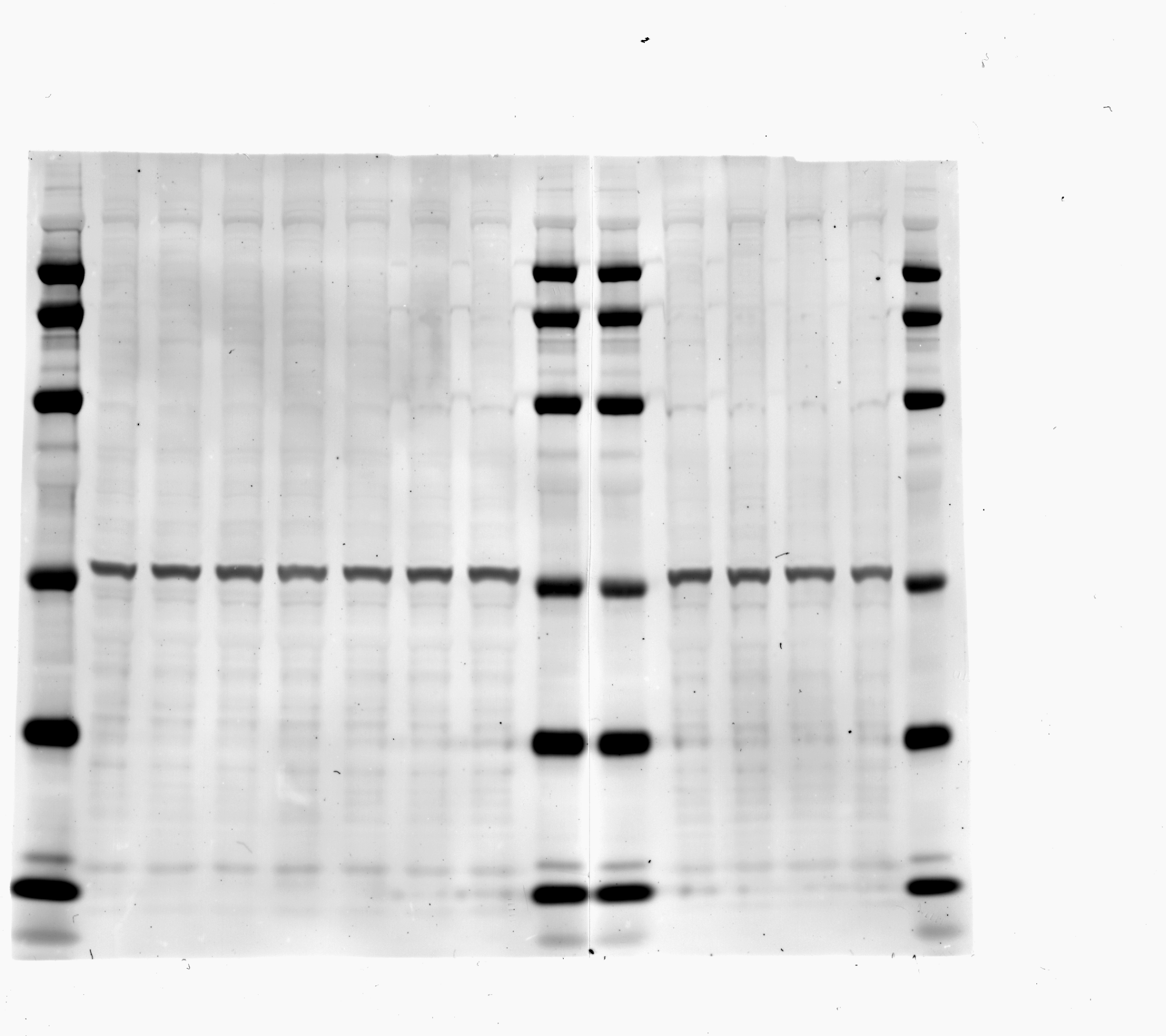

Supplement: Source data 1. [file elife-71980-data1.zip › Original and uncropped gel and blot images/Figure 3E-original tubulin blot.png]

**Figure 3E**  
**Uncropped blots with labels**

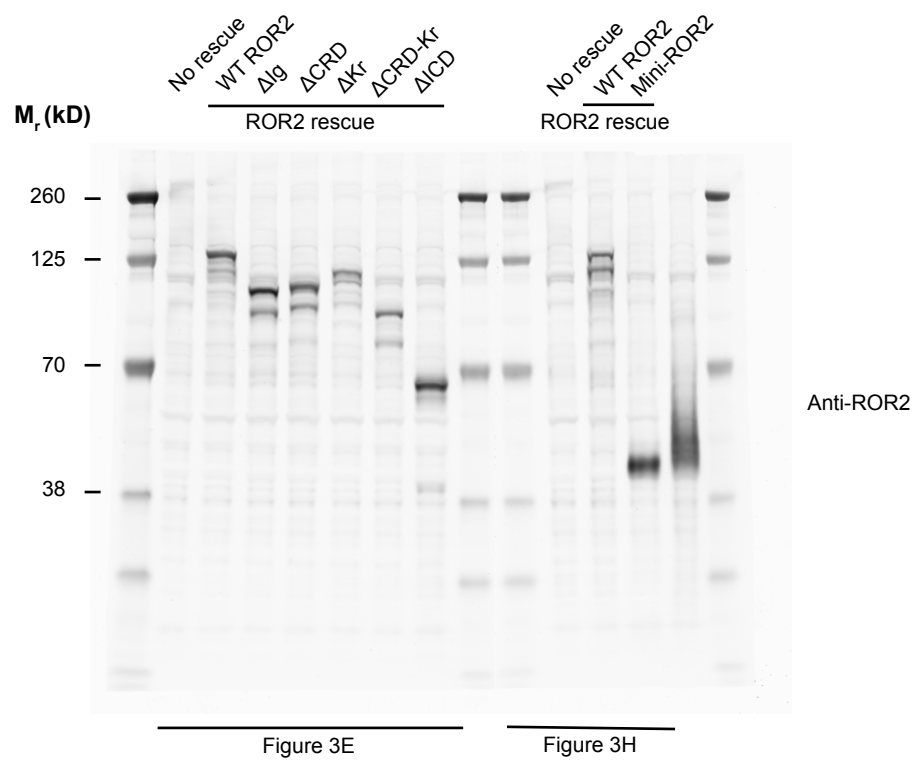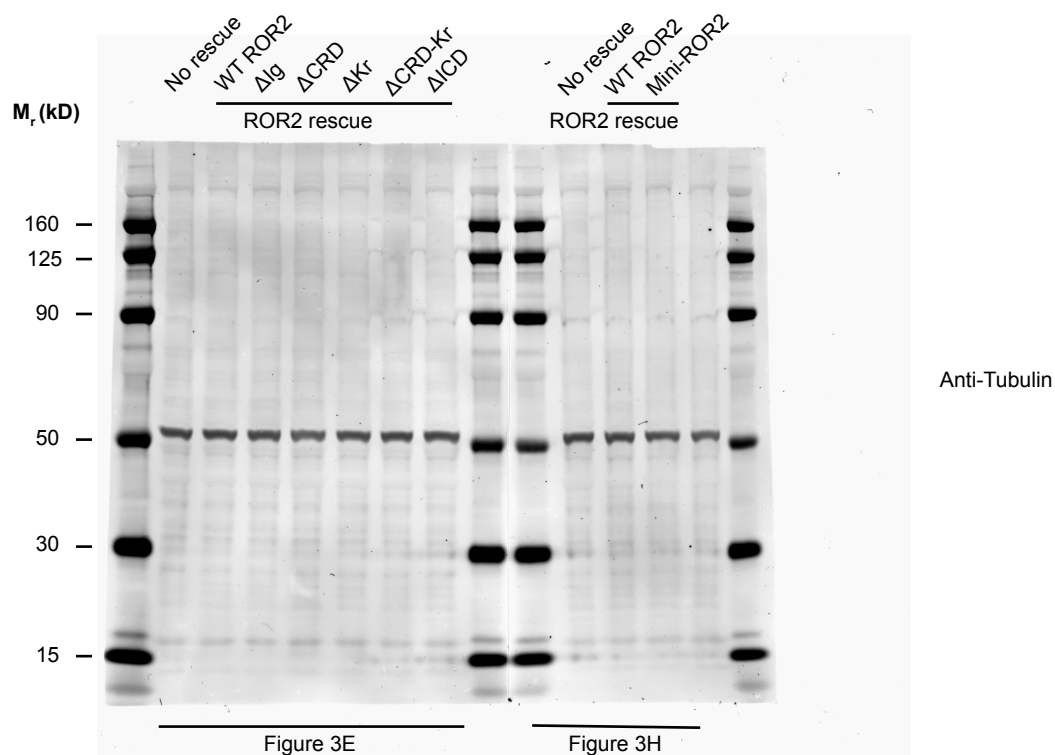

Supplement: Source data 1. [file elife-71980-data1.zip › Original and uncropped gel and blot images/Figure 3E-uncropped blots with lablels.pdf]

Figure 3H  
Uncropped blots with labels

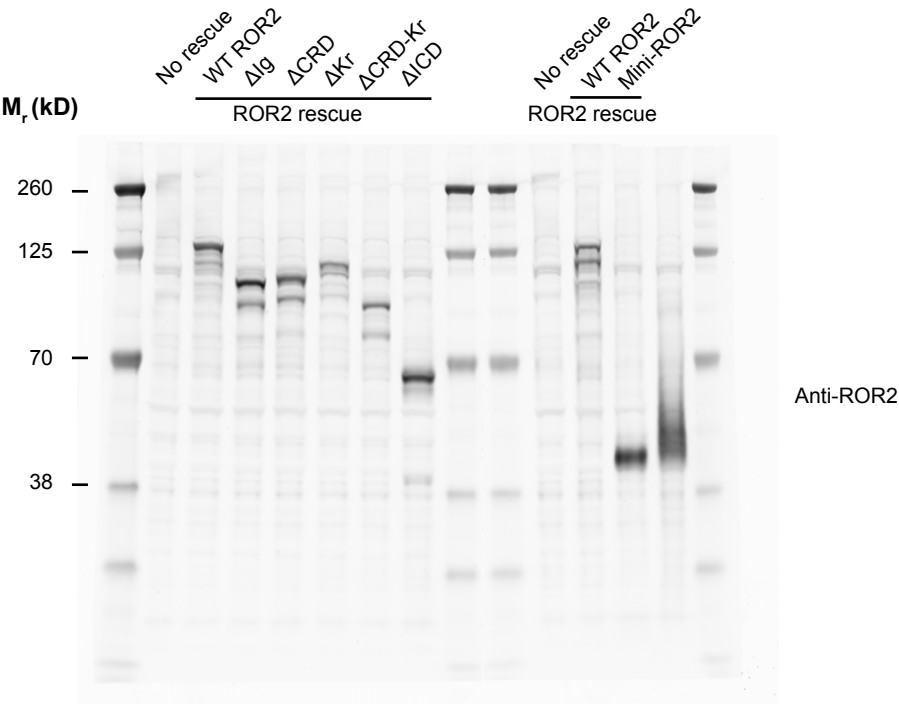

Figure 3E

Figure 3H

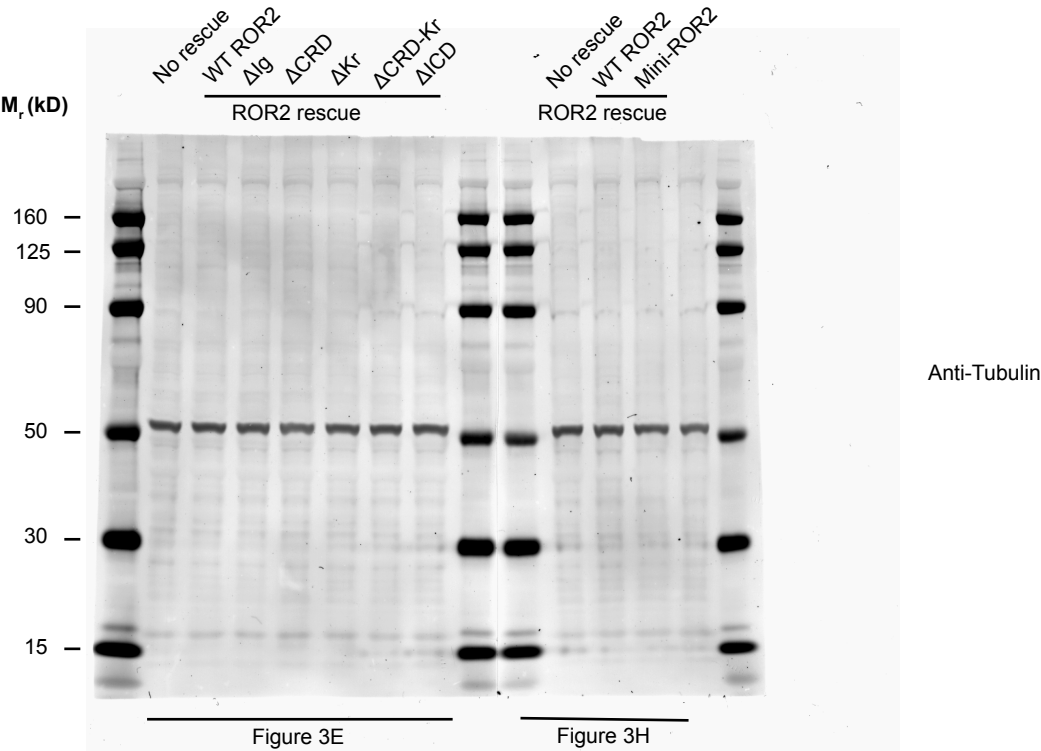

Figure 3E

Figure 3H

Supplement: Source data 1. [file elife-71980-data1.zip › Original and uncropped gel and blot images/Figure 3H-uncropped blots with lablels.pdf]

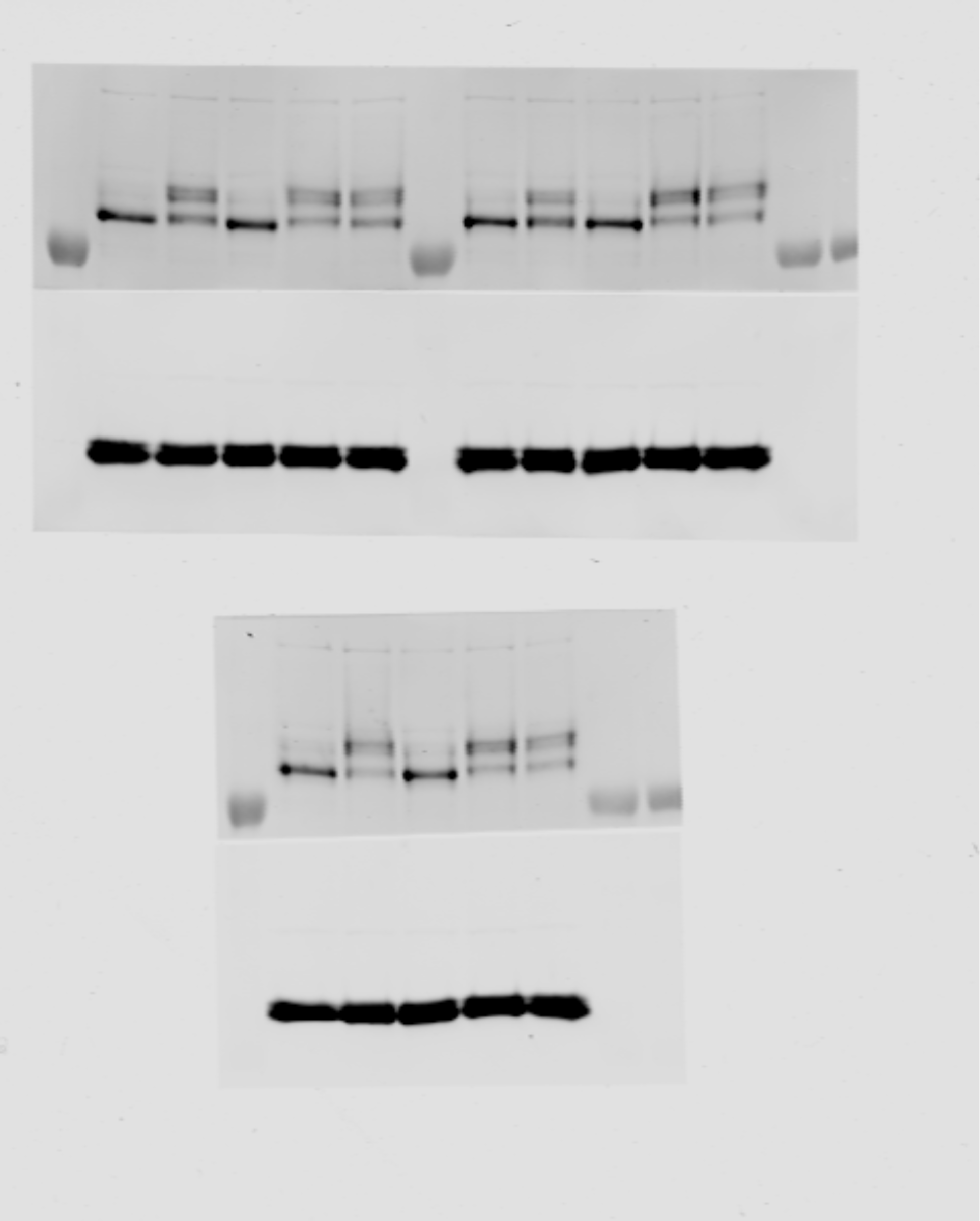

Supplement: Source data 1. [file elife-71980-data1.zip › Original and uncropped gel and blot images/Figure 4-figure supplement-original blots used for DVL2 panels.png]

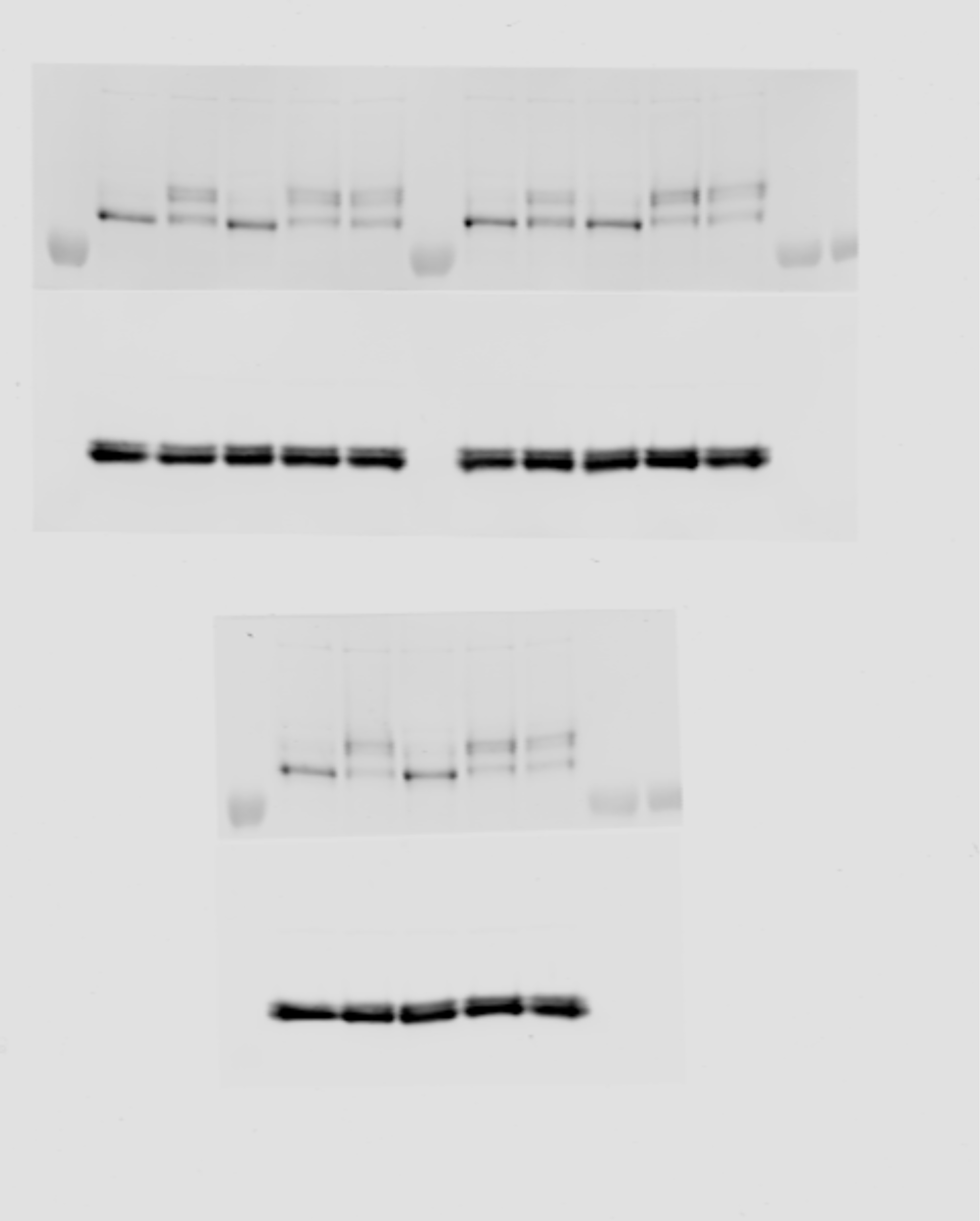

Supplement: Source data 1. [file elife-71980-data1.zip › Original and uncropped gel and blot images/Figure 4-figure supplement-original blots used for tublin panels.png]

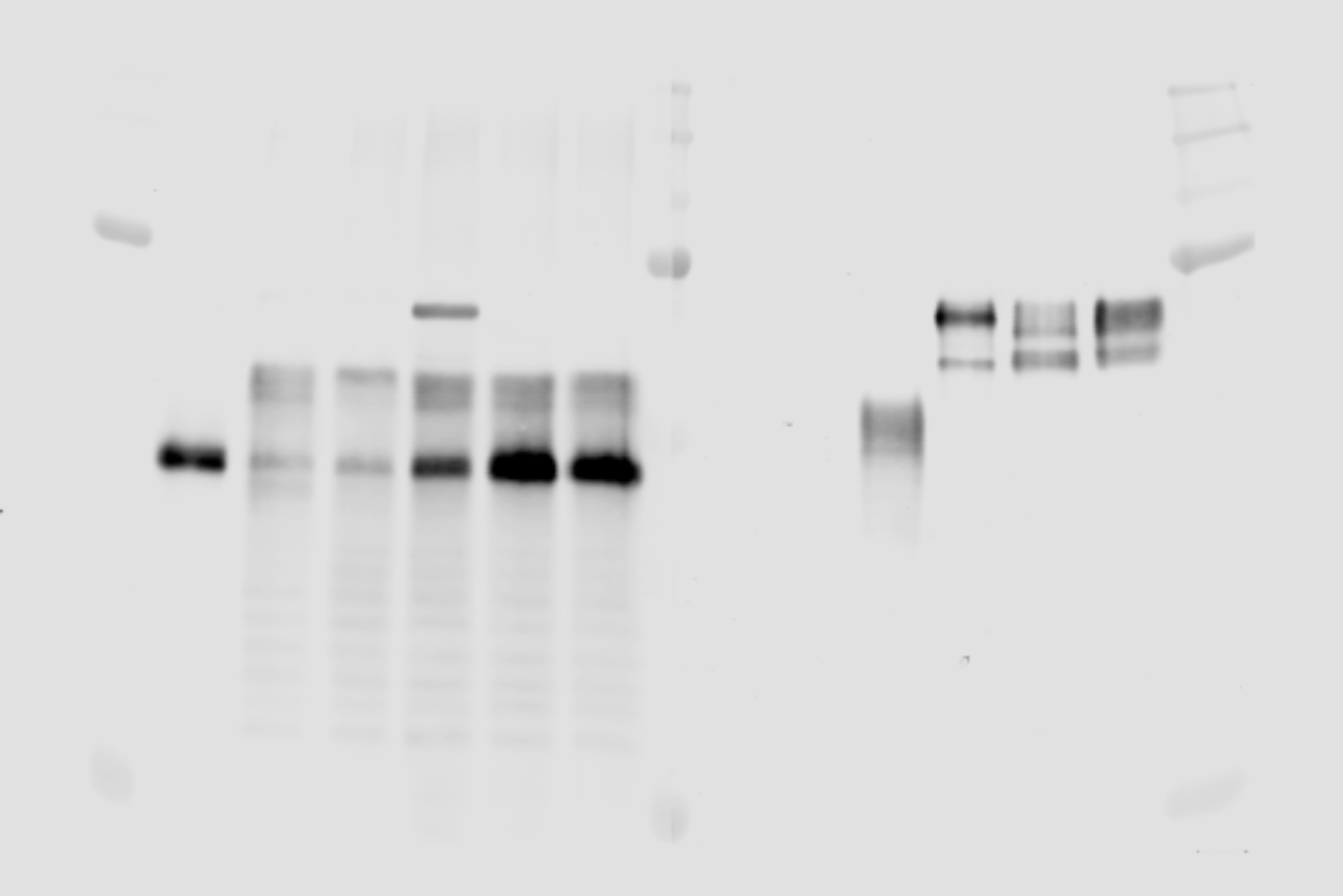

Supplement: Source data 1. [file elife-71980-data1.zip › Original and uncropped gel and blot images/Figure 4A-original Wnt5a and Fc blots.png]

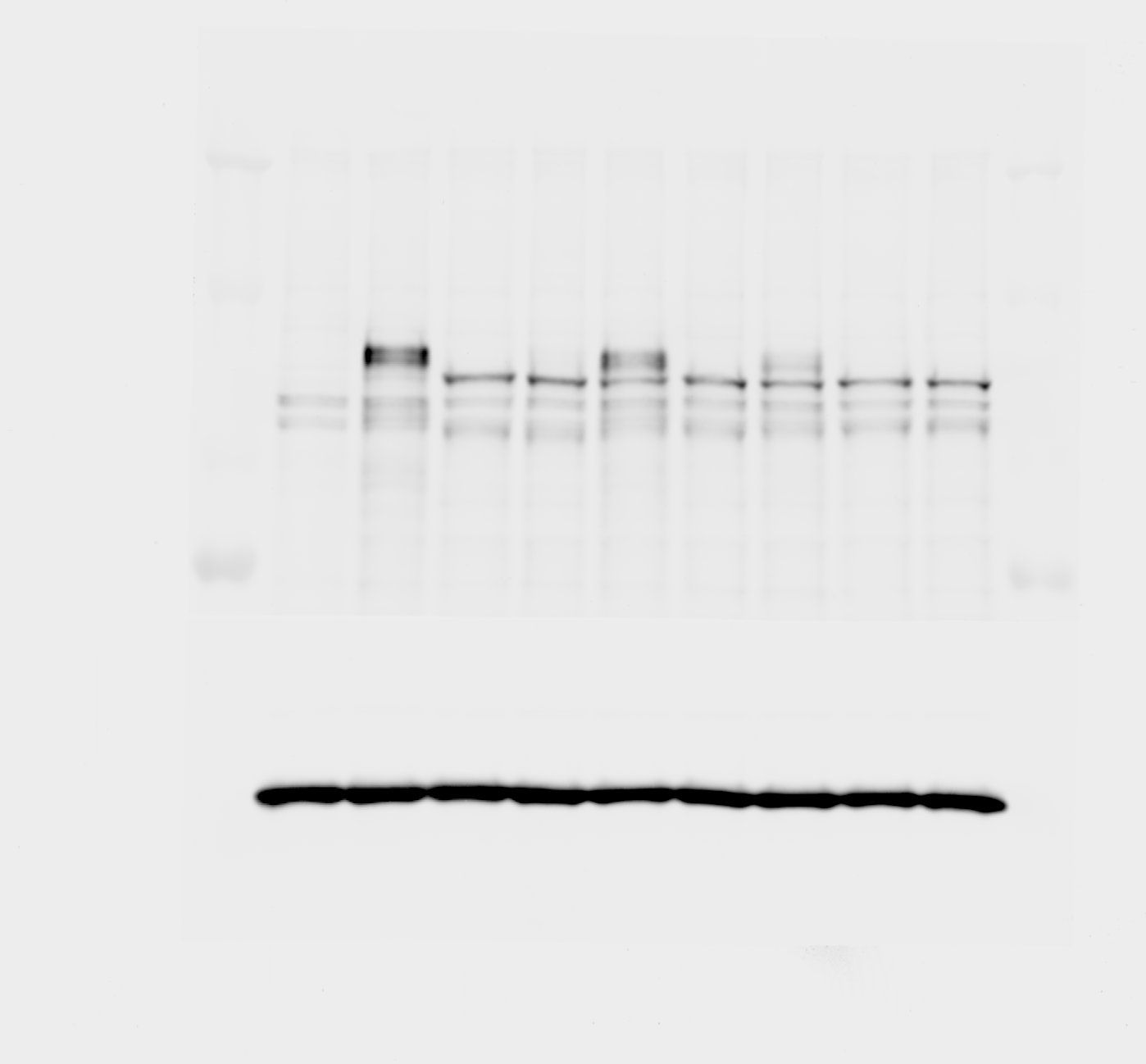

Supplement: Source data 1. [file elife-71980-data1.zip › Original and uncropped gel and blot images/Figure 5A-original Ror2 and tubulin blot.png]

**Figure 5A**  
**Uncropped blot with labels**

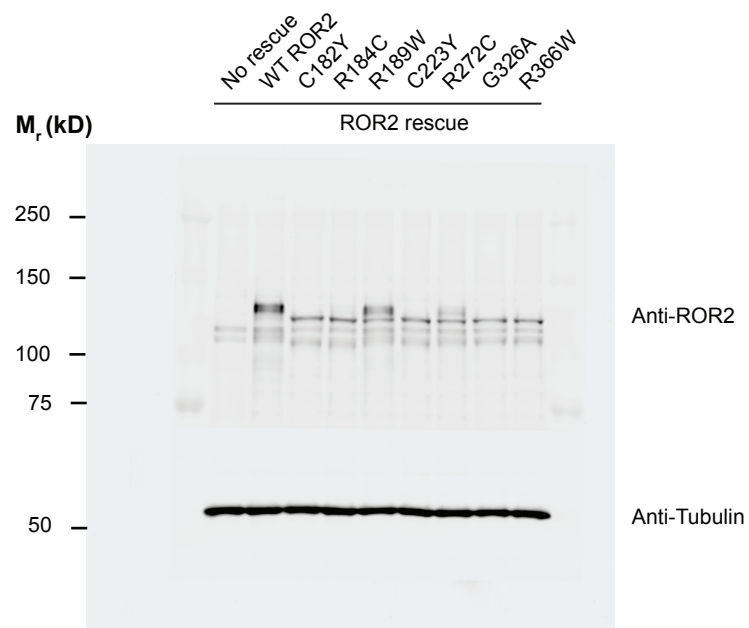

Supplement: Source data 1. [file elife-71980-data1.zip › Original and uncropped gel and blot images/Figure 5A-uncropped blot with labels.pdf]

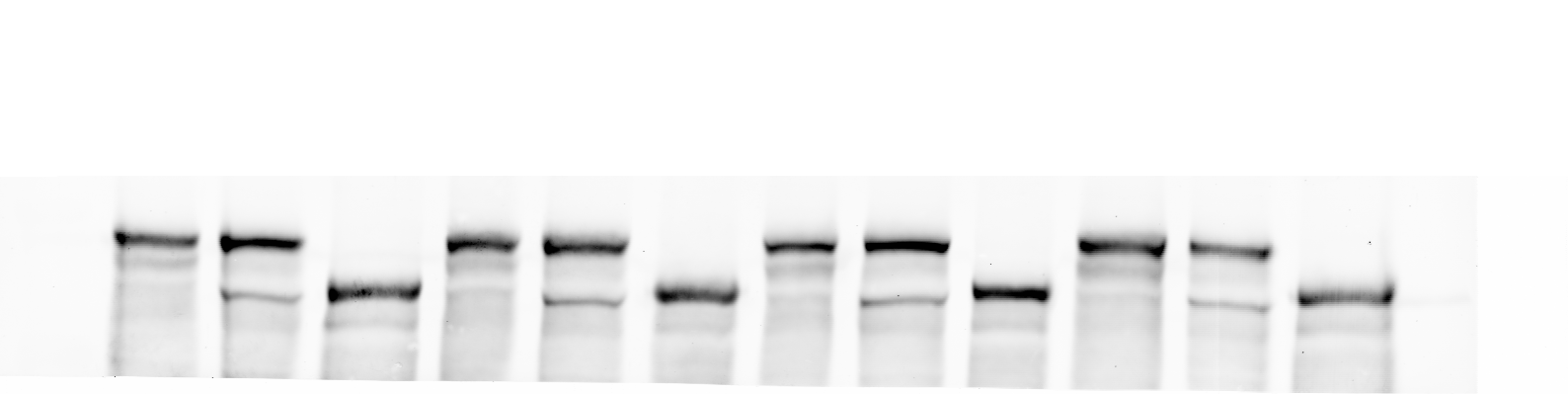

Supplement: Source data 1. [file elife-71980-data1.zip › Original and uncropped gel and blot images/Figure 5C-original Lrp6 blot left side.png]

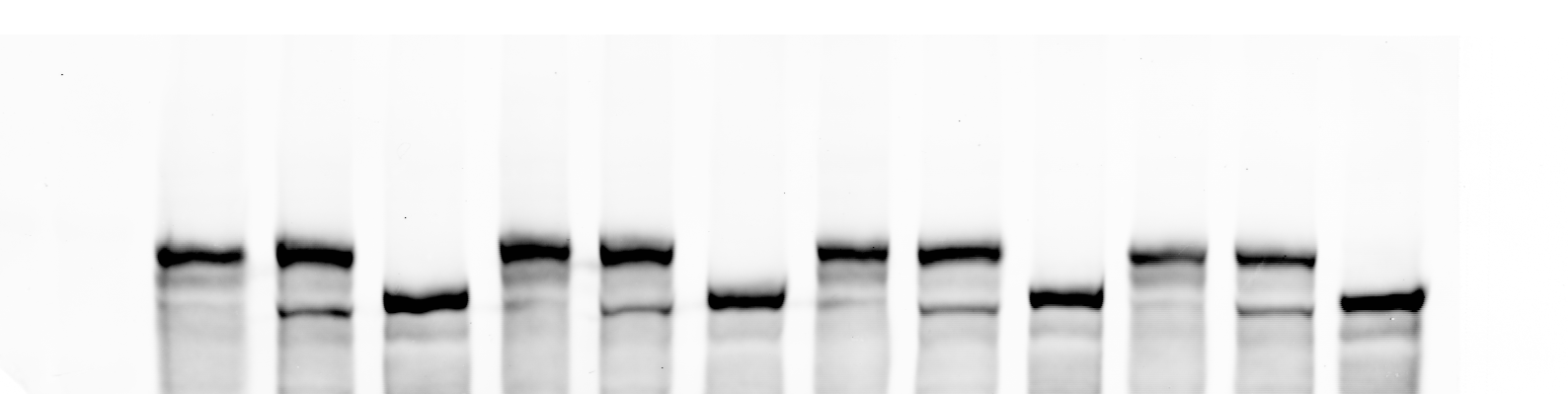

Supplement: Source data 1. [file elife-71980-data1.zip › Original and uncropped gel and blot images/Figure 5C-original Lrp6 blot right side.png]

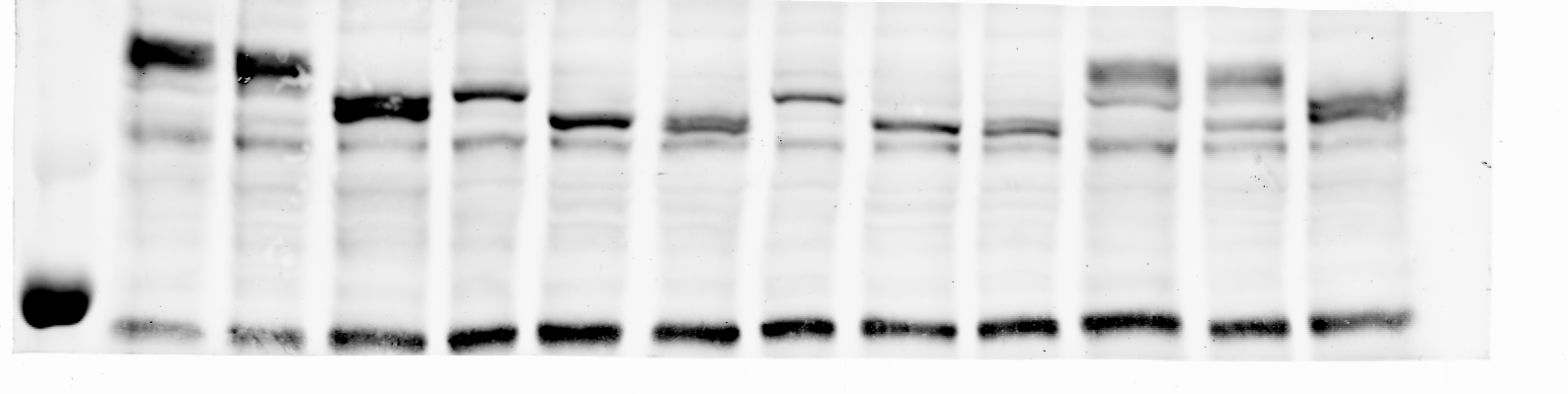

Supplement: Source data 1. [file elife-71980-data1.zip › Original and uncropped gel and blot images/Figure 5C-original Ror2 blot left side.png]

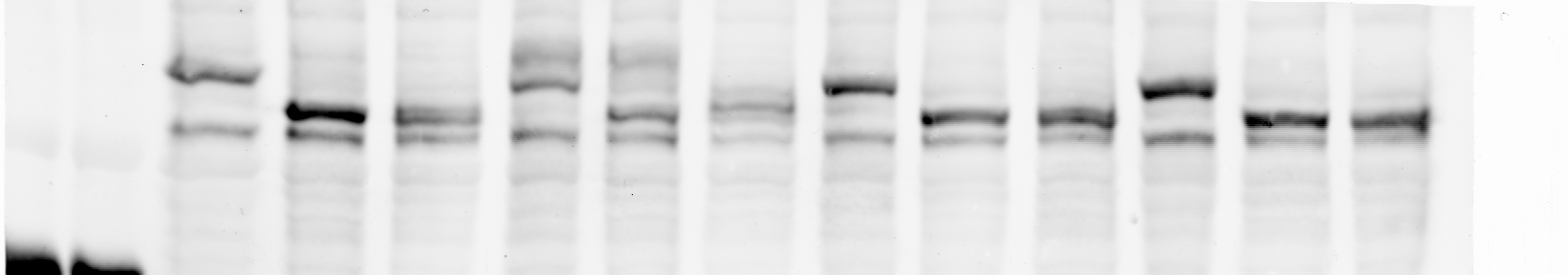

Supplement: Source data 1. [file elife-71980-data1.zip › Original and uncropped gel and blot images/Figure 5C-original Ror2 blot right side.png]

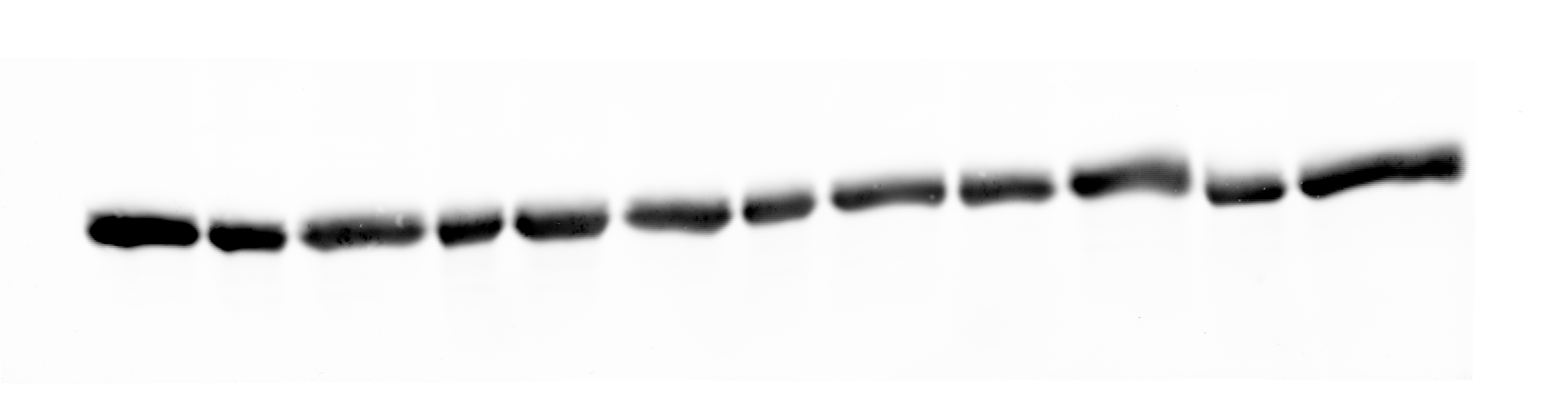

Supplement: Source data 1. [file elife-71980-data1.zip › Original and uncropped gel and blot images/Figure 5C-original tubulin blot left side.png]

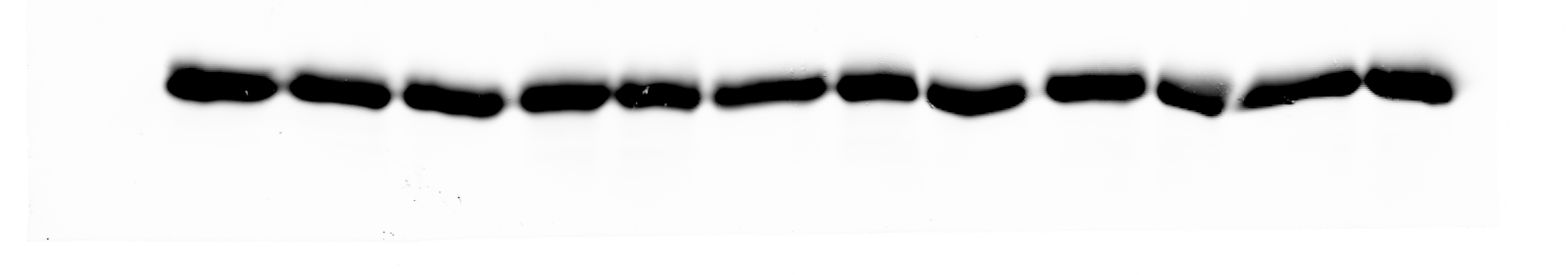

Supplement: Source data 1. [file elife-71980-data1.zip › Original and uncropped gel and blot images/Figure 5C-original tubulin blot right side.png]

Figure 5C  
Uncropped blots with labels

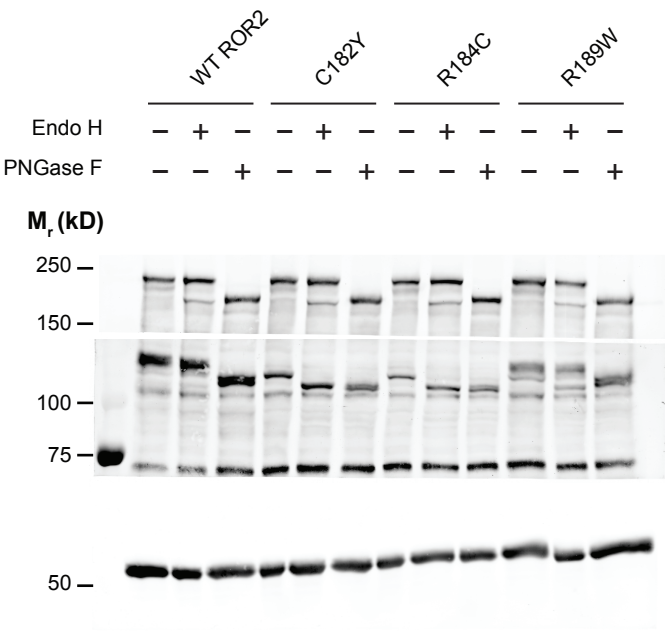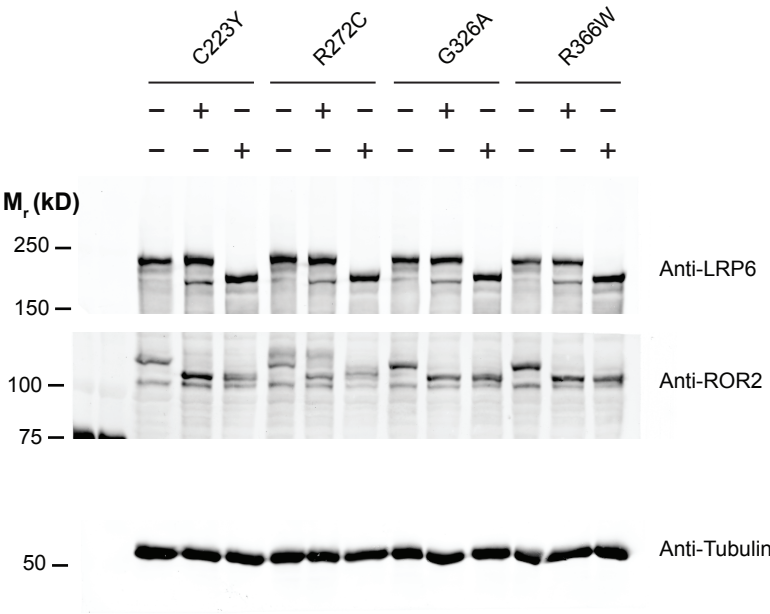

Supplement: Source data 1. [file elife-71980-data1.zip › Original and uncropped gel and blot images/Figure 5C-uncropped blots with labels.pdf]
